# Helsinki experience on nonvitamin K oral anticoagulants for treating cervical artery dissection

**DOI:** 10.1002/brb3.349

**Published:** 2015-05-30

**Authors:** Satu Mustanoja, Tiina M Metso, Jukka Putaala, Noora Heikkinen, Elena Haapaniemi, Oili Salonen, Turgut Tatlisumak

**Affiliations:** 1 Department of Neurology, Helsinki University Central HospitalHelsinki, Finland; 2 Department of Neuroradiology, Helsinki University Central HospitalHelsinki, Finland

**Keywords:** Acute stroke, anticoagulation, cervical arterial dissection

## Abstract

**Background:**

Cervical artery dissection (CeAD) patients with or without stroke are frequently treated with either antiplatelet agents or vitamin K antagonists (VKAs), but few data are reported on the use of nonvitamin K oral anticoagulants (NOACs).

**Methods:**

Between November 2011 and January 2014, we recorded data from patients with a stroke due to vertebral (VAD) or internal carotid artery dissection (ICAD). Patients using oral anticoagulants were included in the study and were divided into two treatment groups: patients using NOACs and those using VKAs. Excellent outcome was defined on modified Rankin Scale (mRS) ≤1 at 6 months.

**Results:**

Of 68 stroke patients (67% male; median age 45 [39–53]), six (8.8%; two with VAD and four with ICAD) were treated with NOACs: three with direct thrombin inhibitor dabigatran and three with direct factor Xa inhibitor rivaroxaban. National Institutes of Health Stroke Scale score at baseline was 4 (3–7) in the NOAC versus 2 (1–7) in the VKA groups. Complete recanalization at 6 months was seen in most patients in the NOAC (*n* = 5; 83%) and VKA (*n* = 34; 55%) groups. All the patients using NOACs had mRS ≤1 at 6 months and none had an intracerebral hemorrhage (ICH). In the VKA group most patients (*n* = 48; 77%) had mRS ≤1, one patient (1.7%) had an ICH and one died.

**Conclusions:**

In this small, consecutive single-center patient sample treating ischemic stroke patients with CeAD with NOACs did not bring up safety concerns and resulted in similar, good outcomes compared to patients using VKAs.

## Background

Cervical arterial dissections (CeAD), that is, vertebral artery (VAD) and internal carotid artery (ICAD) dissections are common etiologies of ischemic stroke in the young (Yesilot Barlas et al. 2013). Early treatment is crucial as it can prevent vessel occlusion or embolic sequels and avoid serious neurologic deficits. Most physicians prescribe anticoagulants for stroke prevention in acute CeAD, although there are no randomized trials comparing the safety and efficacy of anticoagulants with antiplatelets or placebo (Engelter et al. 2007; Sarikaya et al. 2013).

Anticoagulation with nonvitamin K oral anticoagulants (NOACs) is increasingly used for stroke prevention in patients with atrial fibrillation (AF), instead of vitamin K antagonists (VKAs), as both direct factor Xa (Granger et al. 2011; Patel et al. 2011) and direct thrombin (Connolly et al. 2009) inhibitors have been shown to have similar or better safety and efficacy profiles compared with warfarin***.*** There is few data on their use in ischemic stroke patients with CeAD (Caprio et al. 2014); and only one report was found with 10 stroke patients using NOACs as the secondary prevention of ischemic stroke.

## Methods

Between November 2011 and January 2014 we recorded data from consecutive patients with a stroke due to VAD or ICAD. This study was approved by our institutional authorities. Our institutional guidelines recommend the use of anticoagulants in all CeAD patients for 6 months, and the selection of the anticoagulant is decided by the treating neurologist together with the patient.

Patients using oral anticoagulation were included in the study and were divided into two groups: patients using NOACs, and those using VKAs. Patients who underwent endovascular stenting followed by antiplatelet therapy, and patients treated with only heparin or LMWH were excluded. We excluded two patients with multiple traumatic injuries not receiving oral anticoagulation to keep the study population homongenous.

Recurrent ischemic stroke, or intracerebral hemorrhagic (ICH) stroke events, recanalization rate, and functional outcome on the modified Rankin Scale (mRS) within six months were evaluated and compared between the NOAC and VKA-treated groups. An excellent outcome was defined as mRS≤1 at 6 months.

### Statistical analyses

Statistical significance for intergroup differences was assessed by Chi-square test for categorical variables, and Mann–Whitney *U*-test for noncontinuous variables or skewed numerical variables. Statistical significance was set at <0.05. Analyses were performed with SPSS 19 (SPSS Inc., Chicago, IL, USA).

## Results

Of 68 stroke patients included (Table 2004), six were treated with NOACs (three with dabigatran and three with rivaroxaban) and 62 with warfarin. There were slightly more men and VAD was seen in most patients (Table 2014). There were no statistical differences between the two groups in stroke severity, recanalization rate, or outcome (*P *>* *0.05). All the patients using NOACs had mRS ≤1 with no bleeding complications, or recurrent dissections.

**Table 1:** Baseline characteristics and outcome in cervical arterial dissection patients with acute stroke using nonvitamin K oral anticoagulants or vitamin K antagonists

	NOAC (*n* = 6; 8.8%)	VKA (*n* = 62; 91%)	All (*n* = 68)
Age, median (IQR)	44 (38–46)	46 (39–53)	45 (39–53)
Male gender	4 (67)	39 (63)	43 (63)
NIHSS at baseline	4 (2–5)	2 (1–7)	2 (1–6)
Vertebral artery dissection	2 (33)	38 (61)	40 (59)
Occlusion	1 (33)	15 (40)	16 (39)
Stenosis	2 (67)	23 (60)	25 (61)
Internal carotid artery dissection	4 (67)	27 (44)	31 (46)
Occlusion	1 (33)	14 (52)	15 (50)
Stenosis	2 (67)	13 (48)	15 (50)
Symptom onset to hospital	0.5 (0–4.0)	0.5 (0–1.3)	0.5 (0–1.8)
Prior infection	3 (50)	7 (11)	10 (15)
Prior trauma	0	16 (26)	16 (24)
Recanalization	5 (83)	34 (55)	39 (57)
Modified Rankin Scale ≤ 1	6 (100)	48 (77)	54 (79)
Intracerebral hemorrhage	0	1 (1.6)	1 (1.5)
Death	0	1 (1.6)	1 (1.5)

Values are median (interquartile range) and *n* (%). NOAC, nonvitamin K oral anticoagulants; VKA, vitamin K antagonists; NIHSS, National Institutes of Health Stroke Scale. Data on recent infection within 1 week and trauma, physical impact on the head or neck within 1 month were obtained from the patient records.

**Table 2:** Clinical, radiological, and outcome data in six stroke patients with cervical arterial dissection using nonvitamin K oral anticoagulants

Patient/Age^1^/Gender	Baseline NIHSS	Baseline MRA	Recanalization at 6 months	mRS at 6 months
VAD				
1/32/M	2	Intramural hematoma	Yes	0
2/45/M	3	Flame-shaped proximal occlusion	No	0
3/42/M	4	High grade string-like stenosis	Yes	1
ICAD				
4/45/M	6	Intimal flap with proximal occlusion	Yes	0
5/40/M	4	Intramural hematoma and intimal flap with high grade stenosis	Yes	0
6/47/F	1	Intramural hematoma, and intimal flap with high grade stenosis	Yes	0

NIHSS, National Institutes of Health Stroke Scale; MRA, magnetic resonance angiography; mRS, modified Rankin Scale; VAD, vertebral artery dissection; ICAD, internal carotid artery dissection; M, male, F, female.

1 Years.

## Discussion

Stroke is the most common complication of CeAD, occurring in two thirds of the cases (Lichy et al. 2012). Although the molecular mechanisms are still poorly understood in CeAD (Debette 2014), imaging studies and transcranial ultrasound have suggested that arterial embolism is the main mechanism of stroke (Benninger et al. 2004), explaining why anticoagulation is frequently used. ICH is the most feared and serious complication of the use of oral anticoagulation in the prevention and treatment of strokes. In a meta-analysis it was recently concluded that instead of anticoagulants antiplatelets should be used because they cause less bleeding events (Sarikaya et al. 2013), despite a trend was seen during the first week toward more deaths in the antiplatelet group (Sarikaya et al. 2013).

An excellent outcome was seen in all of our patients in the NOAC group and 77% in the VKA group. In a recent report on NOACs and CeAD, there were more recurrent strokes and radiographic progression of dissection while bleeding complications were less common (Caprio et al. 2014). In our study group, only one death and one serious ICH occurred during the 6-month treatment period, both in the VKA group. Fewer bleeding complications have been seen with the NOACs in stroke prevention in AF in three randomized trials (Connolly et al. 2009; Granger et al. 2011; Patel et al. 2011) and the European Society of Cardiology recommends NOACs in preference to VKA therapy for stroke prevention in patients with AF***.*** In the first report with NOACs and CeAD, there were no major bleeds and 5% minor hemorrhagic complications being equal to the rate in the antiplatelet group (Caprio et al. 2014). We anticipate that the indications for the use of NOACs will be extended over time, when new data on their use in different conditions have accumulated. Recently, another off-label indication for using NOACs was reported, as factor Xa inhibitors showed a similar clinical benefit as VKAs in the treatment of cerebral venous thrombosis in a small study cohort of seven patients (Geisbusch et al. 2014).

CeAD etiology dominates in the younger age groups (Metso et al. 2012), unlike AF with a higher risk for bleeding complications associated with older age (Pancholy et al. 2014). The NOAC plasma concentrations achieved with a given dose vary, depending on absorption, renal function, and other factors that can be problematic with the elderly (Reilly et al. 2014). In the young and socially active CeAD patients, at least those with less severe strokes, many could benefit of NOACs given as a fixed dose without laboratory monitoring. Currently it remains unknown whether there is a single concentration range, where the balance between thrombo-embolic events and bleeding events is optimal for CeAD patients. It could be, however, that in more stable CeAD stroke patients the concentration range can be wider, and that NOACs could serve as a first-line treatment for the relatively short treatment period used in CeAD.

Our study has limitations. It is retrospective, and the number of patients treated with NOACs is small. As there are no randomized controlled trials going on, it adds new information on safety issues on secondary prevention with NOACs in stroke patients with CeAD.

## Conclusion

In this small, consecutive single-center patient sample treating ischemic stroke patients with CeAD with NOACs did not bring up safety concerns and resulted in similar, good outcomes compared to patients using VKAs.
